# CRISPR-Cas9 KO Cell Line Generation and Development of a Cell-Based Potency Assay for rAAV-FKRP Gene Therapy

**DOI:** 10.3390/cells12202444

**Published:** 2023-10-12

**Authors:** Marine Geoffroy, Louna Pili, Valentina Buffa, Maëlle Caroff, Anne Bigot, Evelyne Gicquel, Grégory Rouby, Isabelle Richard, Romain Fragnoud

**Affiliations:** 1 Généthon, 91000 Evry-Courcouronnes, France; 2 Université Paris-Saclay/Université Evry, INSERM, Généthon, Integrare Research Unit, UMR_S951, 91000 Evry, France; 3 Institut de Myologie, Université Pierre et Marie Curie Paris 6, UM76 Univ. Paris 6/U974 UMR7215, CNRS Pitié-Salpétrière-INSERM, UMRS 974, 75000 Paris, France; 4 Atamyo Therapeutics, 91000 Evry, France

**Keywords:** Limb-Girdle Muscular Dystrophy Type 9, Fukutin-related protein (FKRP), CRISPR, knock-out, potency assay, gene therapy, rAAV

## Abstract

Limb-Girdle Muscular Dystrophy R9 (LGMDR9) is a dystroglycanopathy caused by Fukutin-related protein (FKRP) defects leading to the deficiency of α-DG glycosylation, essential to membrane integrity. Recombinant adeno-associated viral vector (rAAV) gene therapy offers great therapeutic promise for such neuromuscular disorders. Pre-clinical studies have paved the way for a phase 1/2 clinical trial aiming to evaluate the safety and efficacy of FKRP gene therapy in LGMDR9 patients. To demonstrate product activity, quality, and consistency throughout product and clinical development, regulatory authorities request several quality controls, including a potency assay aiming to demonstrate and quantify the intended biological effect of the gene therapy product. In the present study, we generated FKRP knock-out (KO) cells fully depleted of α-DG glycosylation using CRISPR-Cas9 to assess the functional activity of a rAAV-FKRP gene therapy. We then developed a high-throughput On-Cell-Western methodology to evaluate the restoration of α-DG glycosylation in KO-FKRP cells and determine the biological activity of the FKRP transgene. The determination of the half maximal effective concentration (EC_50_) provides a method to compare the rAAV-FKRP batch using a reference standard. The generation of KO-FKRP muscle cells associated with the high-throughput On-Cell-Western technique may serve as a cell-based potency assay to assess rAAV-FKRP gene therapy products.

## 1. Introduction

Dystroglycanopathies represent a group of rare genetic disorders characterized by a defect in the glycosylation process of alpha-dystroglycan (α-DG) [1]. Located on the membranes of the skeletal muscle, heart, brain, and eye tissue, α-DG plays a key role in the interaction between the extracellular matrix and the actin cytoskeleton, crucial for membrane integrity [2,3,4]. The maturation of α-DG at the membrane is governed by several processes in the endoplasmic reticulum and the Golgi apparatus [5]. More than 10 enzymes have been identified as glycosyltransferases able to post-translationally modify α-DG [6].

Among these enzymes, Fukutin-related protein (FKRP) contributes to the glycosylation of α-DG via the transfer of ribitol-5-phosphate (5-Rbo5P) to the long glycan chain in the Golgi apparatus [7,8]. Genetic changes in the FKRP gene lead to the development of a wide spectrum of pathologies, ranging from severe diseases such as Congenital Muscular Dystrophy Type 1C (MDC1C), Walker–Warburg Syndrome (WWS), and Muscle–Eye–Brain diseases (MEB) to mild muscular dystrophies such as Limb-Girdle Muscular Dystrophy Type R9 (LGMDR9) [9,10,11]. Among FKRP-related disorders, LGMDR9 (OMIM#607155), a recessive autosomal dystrophy affecting 1–9/100,000 persons, is caused by mutations in the FKRP gene. Several mutations, such as 826C > A (L276I) and 1343C > T (P448L), lead to the production of the FKRP mutant protein [12] and consequently to a reduction in α-DG glycosylation. The FKRP protein is a transmembrane protein localized in the medial and trans-Golgi apparatus. Depending on the mutation, the FKRP mutant protein is retained in the endoplasmic reticulum and degraded by the proteasome or localized in the Golgi apparatus with functional impairment [12,13]. The disease progresses gradually and begins with muscle atrophy of the shoulder and the pelvic girdles. The symptoms appear around late childhood or early adulthood and are heterogenous, ranging from temporary fatigability to the loss of ambulation up to severe cardiac and respiratory issues for some patients [10,14,15]. To date, despite the relative efficiency of ribitol-5-phosphate therapy illustrated by the orally bioavailable BBP-418 small drug currently under investigation in a phase 2 clinical trial (NCT05775848), no curative treatment is available for patients suffering from this pathology.

FKRP gene therapy represents one of the most promising therapeutic strategies to treat LGMDR9 [16]. Delivered by a recombinant adeno-associated virus (rAAV) vector, the sequence coding for the human full-length FKRP protein can be systemically distributed to the FKRP-deficient patient’s muscles [17,18,19,20]. Pre-clinical studies [20] have paved the way for a phase 1/2 clinical trial sponsored by Atamyo Therapeutics/Généthon (NCT05224505), aiming to evaluate the safety and efficacy of a FKRP gene therapy product (GNT0006/ATA-100) in LGMDR9 patients. To demonstrate the product activity, quality, and consistency of a gene therapy produced throughout product and clinical development, regulatory authorities such as the Food and Drug Administration (FDA) and the European Medicines Agency (EMA) recommend several quality controls [21]. The cell-based potency assay aims to measure the biological activity of the gene therapy product using quantitative biological assays, reflecting the mechanism of action (MoA) of the therapeutic product. Despite limitations in determining an adequate and relevant in vitro experimental model [22], making the development of a cell-based potency test challenging, there is an urgent and unmet need for a relevant and robust in vitro model to assess the restoration of biological activity after drug treatment in LGMDR9 disease.

Previous works have described the glycosylation of α-DG as one of the main MoAs of the FKRP protein [23,24]. While regulatory authorities request mandatory comparative biological activity measurements of rAAV-FKRP batch-to-batch, such an MoA can serve as a scientific endpoint for the development of a robust in vitro potency assay to assess FKRP gene therapy products. Whereas the complete absence of α-DG glycosylation will provide the appropriate dystrophic context to generate a clear assessment of the restoration of the glycosylated α-DG mediated by the rAAV-FKRP, residual α-DG glycosylation was noted in LGMDR9 patient cells [12]. Indeed, an FKRP-null phenotype, causing major defects in the development of the central nervous system, is lethal at the embryonic stage [9], explaining the expression of a presumably partially functional protein in LGMDR9 patient cells. The presence of hypoglycosylated α-DG in patient cells may potentially prevent the clear observation of α-DG glycosylation restoration after rAAV-FKRP treatment. In recent years, the clustered regularly interspaced short palindromic repeats (CRISPR) and CRISPR-associated (Cas) protein systems (CRISPR-Cas9) have emerged as a revolutionary and precise gene editing avenue with the potential to permanently and precisely edit any DNA locus [25,26]. As a powerful and versatile tool, the CRISPR-Cas9 system was previously used to generate innovative knock-out (KO) cell lines to study pathological phenotypes and evaluate the efficacy of potential therapeutic solutions [27,28].

**Figure 1:**
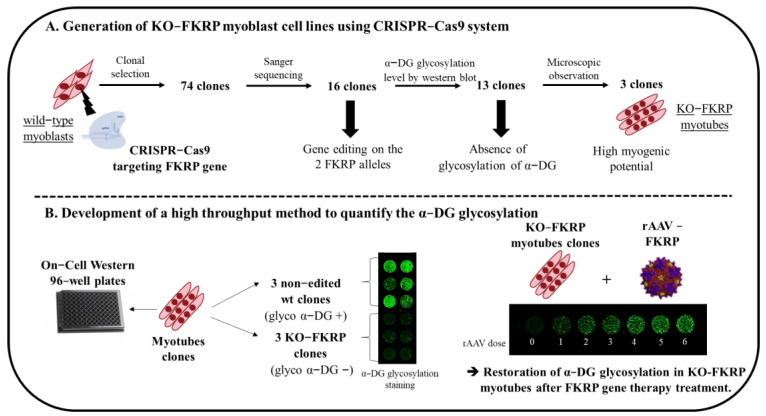
Schematic representation of the generation of KO-FKRP cell lines and the development of a high−throughput method to quantify α-DG glycosylation. (**A**) Strategy to generate KO−FKRP myoblasts using the CRISPR−Cas9 system and to select KO−FKRP clones. (**B**) Development of a high−throughput method to quantify α−DG glycosylation after FKRP gene therapy treatment.

In the present study, we describe the generation of a KO-FKRP immortalized myoblast line fully depleted of α-DG glycosylation using the CRISPR-Cas9 system to develop a robust cell-based potency assay to assess FKRP gene therapy products (Figure 1). The CRISPR-Cas9 gene editing was performed by targeting the translation initiation site (TIS) of the human FKRP gene. Following clonal selection, we isolated and analyzed 74 initial clones harboring the expected gene editing to finally select three KO-FKRP clones with high myotube differentiation potential. Based on the KO-FKRP myoblast cell lines generated, we next developed an innovative in vitro potency assay combining the quantitative measurement of an immunofluorescence signal such as in a Western blot assay and the high-throughput capacity of an ELISA assay [29,30]. Overall, we developed an “On-Cell Western” method allowing the quantification of the biological function of the GNT0006/ATA-100 FKRP gene therapy product in a dose-dependent manner. This study may support the use of this high-throughput technique as a cell-based potency assay for FKRP gene therapy products.

## 2. Materials and Methods

### 2.1. Cell Culture and Differentiation

Human wild-type (wt) AB1190 cells are immortalized myoblasts established from muscular biopsies of healthy individuals, and FKRP 17PV cells are human immortalized myoblasts established from human LGMDR9 muscular biopsies characterized by a compound heterozygous FKRP mutation (c.899T > C and c.798_819dup/p.Val300A). Both cell lines were obtained from the MyoLine immortalization platform of the Myology Institute [31]. KO-FKRP myoblasts were generated from AB1190 cells. Both cells were maintained in the growth medium, Skeletal Muscle Cell Growth Medium (Promocell, Heidelberg, Germany, C-23060) supplemented with 12.5% bovine serum albumin (Eurobio, Les Ulis, France, CVFSVF00-01) at 37 °C in 5% CO_2_. Cells were seeded on 6-well plates (2.9 × 10^5^/well) or 96-well plates (9.6 × 10^4^/well), respectively, for Western blot/Simple Western and On-Cell Western, pre-coated with 3 mg/mL of collagen I bovine (StemCell Technologies, Vancouver, BC, Canada, #04902). After 48 h, myoblasts were differentiated using Skeletal Muscle Cell Differentiation Medium (Promocell, C-23061). After 4 days of differentiation, myotubes were collected for different analyses.

### 2.2. Generation of KO-FKRP Cell Line

Human wt AB1190 myoblasts were seeded (50,000 cells/well) in 24-well plates pre-coated with 3 mg/mL of collagen bovine I (StemCell Technologies, #04902) and transfected 24 h later using the CRISPR Gene Knockout Kit V2 specific to the human FKRP gene (Synthego, Redwood City, CA, USA), based on the protocol provided by Synthego: “CRISPR Editing of Immortalized Cell Lines with RNPs Using Lipofection for 24-Well Plates” (2021). The kit provided a mix of 3 sgRNAs targeting DNA regions near the translation initiation site (TIS) of the FKRP gene (CTGGCAGCGGGTGAGCCGCA, AGCCTAGGAATTCCCGGGCC, GCACCAGGACGGTGACACGG). Ten pmol of the sgRNA mix (kit from Synthego) and 10 pmol of Streptococcus Pyogenes Cas9 (spCas9) (Synthego) were transfected using Lipofectamine CRISPRMAX (Thermo Fisher Scientific, Waltham, MA, USA, CMAX00008) following the manufacturer’s protocol. Two days later, after myoblasts’ trypsinization, cells were suspended in growth medium supplemented by CloneR 1X (StemCell Technologies, #05888) to increase single-cell survival. Then, serial dilutions were performed in 96-well plates to obtain 1 cell/well for clonal selection. Each well was observed under a microscope (Invitrogen, Carlsbad, CA, USA, 4471136) to select those containing a single-cell clone. These picked clones were amplified and sequenced using the Sanger sequencing method.

### 2.3. DNA Analysis by Sanger Sequencing

Genomic DNA from edited cells was extracted using QuickExtract™ DNA Extraction Solution (Lucigen, Middleton, WI, USA) following the protocol supplied. Fifty ng of genomic DNA was used to amplify the area surrounding the cutting site of the sgRNAs by polymerase chain reaction (PCR) using the KAPA2G Fast ReadyMix kit (Roche, Basel, Switzerland). Primers used for the amplification were 5′-TGGAGAAGGGGAAGTCAAAGC-3′ and 5′-TGGAGCAGCGCCAGAC-3′. PCR was performed with the Bio-Rad C1000 Touch™ Thermal Cycler. PCR products were visualized on a 2% agarose gel and then sent for Sanger sequencing (Genewiz, Leipzig, Germany). The percentage of insertions and deletions (InDels) was calculated using the TIDE (https://tide.nki.nl/ accessed on 4 July 2023) and ICE (https://ice.synthego.com/#/ accessed on 4 July 2023) software programs. Off-target analyses were performed using the CRISPOR software (http://crispor.tefor.net/ accessed on 4 July 2023) and predicted by the CFD specificity score [32].

### 2.4. Immunofluorescence Staining

Myoblasts or myotubes were fixed with PFA 4% and permeabilized with 0.5% Triton X-100. After the blocking of non-specific binding, cells were incubated overnight at 4 °C with primary antibodies, washed three times with PBS, and incubated for one hour with a secondary antibody at room temperature. The antibodies used were anti-MyHC monoclonal antibody (MF-20, Developmental Studies Hybridoma Bank, Iowa City, IA, USA, 1:10), revealed with Alexa Fluor 594-conjugated anti-mouse IgG, and anti-alpha Actinin 2 recombinant rabbit monoclonal antibody (7H1L69, ref 701914, 1:100), revealed with Alexa Fluor 488-conjugated anti-rabbit IgG. Nuclei were counterstained with DAPI. The fusion index was calculated as the ratio between the number of nuclei inside MyHC-positive myotubes and the total number of nuclei present in a field (six fields counted per condition).

### 2.5. AAV Vectors and Transduction

A codon-optimized form of the human coding FKRP sequence (1488 bp) was synthetized (Genescript, Piscataway, NJ, USA) and inserted directly into an AAV-based pSMD2-derived vector carrying type 2 inverted terminal repeats (ITRs) to obtain the plasmid pAAV-FKRP. The expression of the FKRP transgene is driven by the human muscle-specific desmin promoter. The FKRP sequence is followed by a 22 bp miRNA-208a sequence target (miR-208a-T) and by the HBB2 (hemoglobin subunit β2) polyadenylation signal. 

Adenovirus-free rAAV2/9 viral preparations were generated by packaging AAV2-ITR recombinant genomes in AAV9 capsids, using a three-plasmid transfection protocol, as previously described [33]. Briefly, HEK293 cells were co-transfected with pAAV-FKRP, a RepCap plasmid (pAAV2.9, Dr J. Wilson, UPenn, Philadelphia, PA, USA), and an adenoviral helper plasmid (pXX6 [34]) at a ratio of 1:1:2. Viral genomes were quantified by a TaqMan real-time PCR assay using primers and probes corresponding to the gene of interest. The primer pairs and TaqMan probes used for amplification were as follows. Primer forward: 5′-GCCCTTCTACCCCAGGAATG-3′; primer reverse: 5′-AAACTTCAGTCCAGGAACCTC-3′; TaqMan probe: 5′-TGCCCTTTGCTGGCTTTGTGGCCCAGGC-3′. The AAV titer of the AAV9-FKRP used in this study was 8.5 × 10^13^ VG/mL.

For transduction, the KO-FKRP myoblasts were seeded and then directly transduced by adding the AAV9-FKRP vector into the wells, at different multiplicity of infection (MOI) doses. After two days, differentiation was induced by media replacement.

The range of AAV transduction was 500 to 160,000 MOI. The MOI calculation was based on the number of myoblasts on the day of the seeding. 

### 2.6. Western Blot

Four days after cell differentiation, the myotubes were scraped on ice and the proteins extracted using a mix of RIPA lysis buffer (Thermo Fisher Scientific, 89901) and a protease inhibitor cocktail (PIC) (Roche, 05 892 791 001). Samples were heated at 100 °C for 5 min and then centrifuged at 13,000 rpm for 15 min. The supernatant containing the proteins was collected, prepared with Laemmli/DTT (Bio-Rad, Hercules, CA, USA, #1610747), and denatured at 95 °C for 5 min. Proteins were separated on Any kDa Mini-PROTEAN TGX gels (Bio-Rad, #4569034) in 1X Tris-Glycine SDS migration buffer for 1.5 h at 120 V. Subsequently, proteins were transferred into nitrocellulose membranes using the Trans-Blot Turbo RTA Mini 0.2 µm Nitrocellulose Transfer Kit in the Trans-Blot^®^ Turbo™ Transfer System (Bio-Rad). Membranes were saturated for 2 h with Intercept Blocking Buffer (PBS) (LI-COR^®^, Lincoln, USA, 927-70001) and probed with antibodies against glycosylated α-DG IIH6C4 (Millipore, Burlington, MA, USA, 05-593-I, 1:1000 dilution and DSHB, 1:50 dilution), anti-DG core (R&D Systems, Minneapolis, MN, USA, AF6868, 1:1000 dilution), and antibody against β-DG (DSHB, MANDAG2 (7D11), 1:1000 dilution) overnight at 4 °C. The following day, LI-COR^®^ infrared-based secondary antibodies (1:3000 dilution) were incubated on the blots for 1 h at room temperature. Protein bands were revealed and quantified by infrared imaging using the Odyssey^®^ CLx Imaging System (LI-COR^®^ Biosciences).

### 2.7. JESS Simple Western™

Four days after cell differentiation, the myotubes were harvested by trypsinization and the proteins extracted using a mix of RIPA lysis buffer supplemented by PIC. A total colorimetric assay of the proteins contained in the samples was carried out using the BCA Protein Assay Kit (Thermo Fisher Scientific, 23227). To perform the Simple Western™ assay, the samples, reagents, antibodies, and plate were prepared following the 12–230 kDa Separation Module (ProteinSimple^®^, Bio-Techne, Minneapolis, MN, USA). Briefly, samples were diluted to the optimized concentration of 1 mg/mL in 1X fluorescent master mix (EZ standard pack I; ProteinSimple^®^, PS-ST01EZ-8) and denatured at 95 °C for 5 min. The monoclonal mouse IgG1 antibody anti-FKRP (Santa-Cruz, Dallas, TX, USA, Sc-374642) was used at 1:10 and the polyclonal rabbit antibody anti-alpha Tubulin (Abcam, Cambridge, UK, ab4074) was used at 1:1000, diluted in Antibody Diluent 2 (Bio-Techne, 042-203). The secondary antibodies, anti-mouse HRP (DM-001) and anti-rabbit HRP (DM-002), were used according to the Anti-Mouse and Anti-Rabbit Detection Module kit’s instructions (ProteinSimple^®^, Bio-Techne).

### 2.8. On-Cell Western

Cells were seeded in 96-well plates for On-Cell Western™ assays (LI-COR^®^, 926-19156). On-Cell Western™ assays were performed according to the protocol provided by LI-COR^®^. Four days after cell differentiation, myotubes were fixed with 4% paraformaldehyde for 10 min. Each well was blocked with Intercept^®^ (TBS) Blocking Buffer (LI-COR^®^, 927-60001) for 2 h at room temperature. Primary antibodies were incubated overnight at 4 °C. Mouse monoclonal anti-α-Dystroglycan IIH6C4 antibody (Millipore, 05-593-I) was first used at 1:100, and mouse monoclonal anti-β-Dystroglycan was used at 1:100 (DSHB, MANDAG2 (7D11)). The following morning, after several TBS washes, the secondary antibodies were incubated for 1 h. IRDye^®^ 800CW donkey anti-mouse IgG secondary antibody and IRDye^®^ 680RD donkey anti-rabbit IgG secondary antibody were used at 1:800. 

### 2.9. Statistics

Results were analyzed using Prism (GraphPad Software Inc., La Jolla, CA, USA). The comparison of cell groups for the fusion index was performed using one-way analysis of variance (ANOVA) with the post-hoc Tukey test. The determination of statistical differences between two groups was performed using Student’s t-test with a two-tailed distribution, assuming equal or unequal sample variance depending on the equality of the variance. Data are presented as the mean ± SEM, with n indicating the number of independent biological replicates used in each group. Differences were considered significant at (*) *p* < 0.05; (**) *p* < 0.01; (***) *p* < 0.001; and (****) *p* < 0.0001.

## 3. Results

### 3.1. Generation of KO-FKRP Myoblasts by CRISPR-Cas9 System

**Figure 2:**
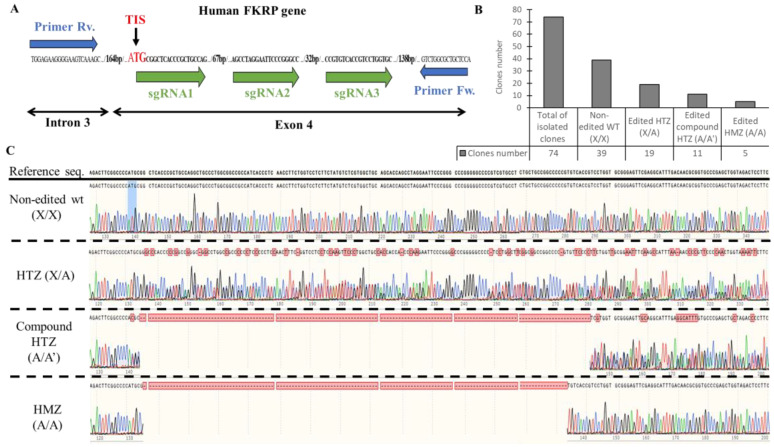
Generation of KO-FKRP cell lines and characterization by Sanger sequencing. (**A**) CRISPR-Cas9 editing and sequencing strategies: three sgRNAs (sgRNA1, sgRNA2, and sgRNA3) were transfected in wt myoblasts near the translation initiation site (TIS) to edit the FKRP DNA in exon 4. The DNA targeted by the sgRNAs was amplified with forward (Fw.) and reverse (Rv.) primers, located in intron 3 and in exon 4, respectively. The PCR products from each clone were then analyzed by Sanger sequencing. (**B**) The total isolated clones were divided into 4 sub-groups: the non-edited wt clones (X/X), the edited heterozygous (HTZ) clones (X/A), the edited compound HTZ clones (A/A′), and the edited homozygous (HMZ) clones (A/A). The number of clones for each sub-group was calculated from the Sanger sequencing results. (**C**) Representative sequencing chromatograms for each sub-group are illustrated.

To generate a KO of FKRP expression, three sgRNAs were designed to target the human exon 4 (Figure 2A) containing the entire open reading frame (ORF) and including the translation initiation site (TIS) [9]. sgRNA1 is located at the beginning of the TIS and sgRNA2 and 3 are situated 67 bp and 119 bp after sgRNA1, respectively. Briefly, the sgRNAs and the Cas9 nuclease were transfected into AB1190 wild-type (wt) myoblasts and clonal selection was performed after 48 h. DNA from each edited cell clone was extracted, amplified by PCR with primers flanking the targeting site, and analyzed by Sanger sequencing.

Out of 74 clones sequenced, 39 were non-edited and considered as wt, and 35 harbored gene editing at the targeted site (Figure 2B). The edited clones were distributed as follows: 19 were heterozygous (HTZ) (one edited allele copy and one non-edited allele copy), 11 were compound heterozygous (two edited allele copies with different gene editing), and five were homozygous (HMZ) (two edited alleles copies with similar gene editing) (Figure 2C). The InDel profile validated the different sub-groups with 0% of InDels for the non-edited clones, 50% of InDels for the HTZ clones, and 96 and 100% for the compound HTZ and the HMZ clones, respectively (Figure S1).

Overall, based on the Sanger sequencing, 16 clones presented gene editing at the targeted site of the FKRP locus (11 compound HTZ and 5 HMZ) and were selected for further analyses.

### 3.2. Characterization of α-DG Glycosylation in Selected KO-FKRP Clones

Based on the sequencing data, the selected clones were studied by Western blot to estimate the level of glycosylated α-DG, the core α-DG expression, and the FKRP expression.

**Figure 3:**
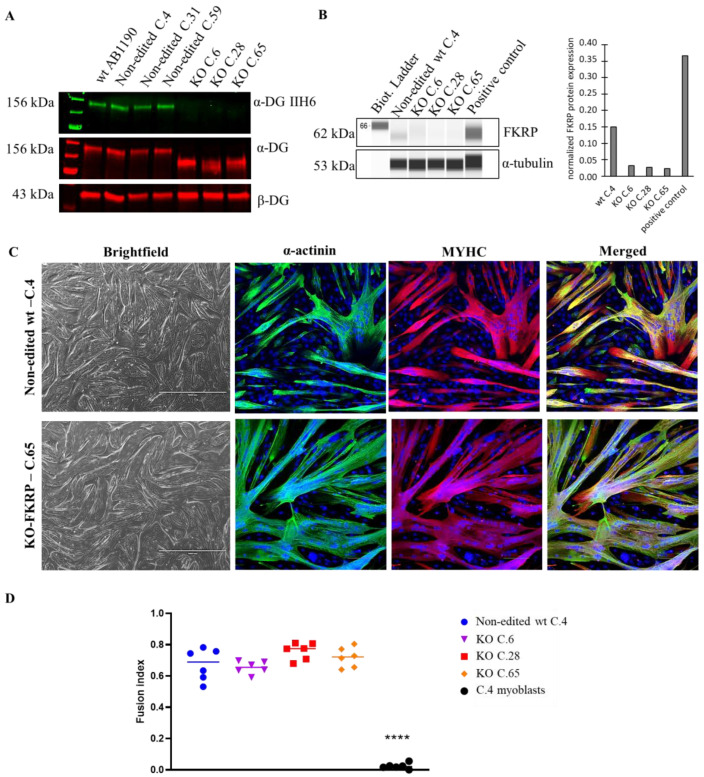
α-DG glycosylation and FKRP expression in KO-FKRP myotubes at the protein level. (**A**) Expression of glycosylated α-DG using IIH6 antibody and of total α-DG by Western blot on the original wt myotubes (wt AB1190), three non-edited wt myotube clones (wt C.4, wt C.31, and wt C.59), and three edited wt myotube clones (KO C.6, KO C.28, and KO C.65). β-DG was used as a housekeeping protein. (**B**) Expression of FKRP protein by Simple Western in a non-edited wt clone (wt C.4) and three edited wt myotube clones (KO C.6, KO C.28, and KO C.65). A sample of myotubes transduced with rAAV-FKRP was used as a positive control. α-tubulin was used as a housekeeping protein. Quantification of FKRP expression, normalized by α-tubulin, was performed. (**C**) Brightfield pictures and immunofluorescence of myogenic markers (α-actinin and MYHC) of one non-edited wt clone (wt C.4) and one edited wt clone (KO C.65) at myotube stage. Cell nuclei were labeled with DAPI dye. Scale bars = 1000 µm for brightfield pictures; 100 µm for immunofluorescence pictures. (**D**) Fusion index for one non-edited wt clone’s myotubes (wt C.4), three edited wt clones’ myotubes (KO C.6, KO C.28, and KO C.65), and a negative control (non-edited wt C.4 myoblasts). **** *p*-value < 0.0001 following a one-way ANOVA.

The level of glycosylated α-DG was null in 13 out of 16 clones compared to the level obtained with the original AB1190 wt myotubes, the non-edited wt clones (clones 4, 31, and 59), and the 17PV patient cell line (Figure S2). Importantly, these latest immortalized myoblasts established from human LGMDR9 patient cells were previously studied and showed a high level of α-DG glycosylation by Western blot (67% compared to wt), highlighting the necessity to create KO-FKRP cell lines (Figure S3). Next, we focused on three edited clones: 6, 28 (compound heterozygous), and 65 (homozygous). These three clones showed no α-DG glycosylation but expressed the core-α-DG (Figure 3A). Interestingly, the core-α-DG was expressed in the edited clones but with a lower molecular weight than in the non-edited case, highlighting the absence or partial post-translational glycosylation on the α-DG protein. We validated the absence of FKRP expression by Simple Western in the edited clones compared to the non-edited cells and a positive control (0.37 for positive control, 0.15 for non-edited cells, and 0.03, 0.03, and 0.02 for KO C.6, C.28, and C.65, respectively) (Figure 3B). Of note, the level of FKRP enzyme in the non-edited cells was very low, as is known and usual for wt samples. Interestingly, the edited clones presented a strong capacity for myotube formation, as observed by immunofluorescence staining, showing a similar fusion index between wt and KO-FKRP clones (Figure 3C,D and Figure S4).

In summary, we selected three edited clones without expression of the FKRP protein and α-DG glycosylation. Importantly, the selected clones demonstrated a strong capacity for differentiation, a crucial criterion to estimate α-DG glycosylation, mainly present in highly differentiated myotubes [35]. Overall, these FKRP-KO cells lines newly generated by CRISPR-Cas9 represent an adequate model to study the restoration of α-DG glycosylation.

### 3.3. Development of On-Cell Western Method to Detect the Glycosylated α-DG

**Figure 4:**
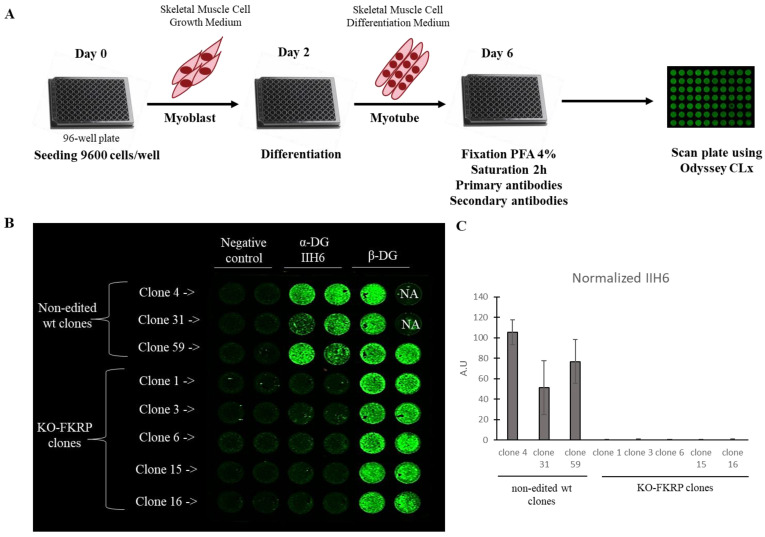
Detection of glycosylated α-DG in KO-FKRP model by On-Cell Western. (**A**) At day 0, wild-type (wt) myoblasts were seeded in 96-well plate at 9600 cells/well in Skeletal Muscle Cell Growth Medium. At day 2, myoblasts were differentiated into myotubes by changing the growth medium to the Skeletal Muscle Cell Differentiation Medium. At day 6, myotubes were fixed with paraformaldehyde (PFA) following saturation and were then incubated with primary antibodies (α-DG IIH6 and β-DG) and secondary antibody. The plate was analyzed using the Odyssey CLx from LI-COR. (**B**) Image of On-Cell Western results showing the expression of glycosylated α-DG (α-DG IIH6) and β-DG in wt clones (clone 4, clone 31, and clone 59) and KO-FKRP (clone 1, clone 3, clone 6, clone 15, and clone 16) myotubes. The green fluorescent staining is representative of the level of protein in each well. β-DG was used as a housekeeping protein. NA (not applicable) corresponds to empty wells. (**C**) Quantification of α-DG IIH6 expression, normalized by β-DG, from wt clones and KO-FKRP clones. Bars are mean ± SEM of n = 2 per condition. A.U. = arbitrary unit. SEM = standard error of the mean.

With the aim of providing a high-throughput approach, we developed an assay based on the On-Cell Western (OCW) technology using the non-edited clones, expressing glycosylated α-DG, and the KO-FKRP clones, lacking the glycosylation of α-DG (Figure 4A). OCW is a variation of the In-Cell Western (ICW) technique, allowing the detection of extracellular targets. Briefly, cells were seeded in a 96-well black plate, and, two days later, myoblasts were differentiated into myotubes for four days. Then, after the fixation of myotubes, the glycosylated α-DG was revealed by the primary antibody IIH6 [4,36], followed by secondary antibody exposure.

In the non-edited clones, α-DG glycosylation was expressed as well as the β-DG protein, as expected (Figure 4B,C). In contrast, in the KO-FKRP clones, no α-DG glycosylation signal was noted, while the expression of the β-DG protein was maintained. The result was confirmed by Western blot (Figure S2).

In conclusion, we developed an innovative OCW high-throughput method, allowing the detection of α-DG glycosylation in a differentiated muscle cell model.

### 3.4. Restoration of Biological Activity in KO-FKRP Clones after Transduction by rAAV-FKRP Vector

**Figure 5:**
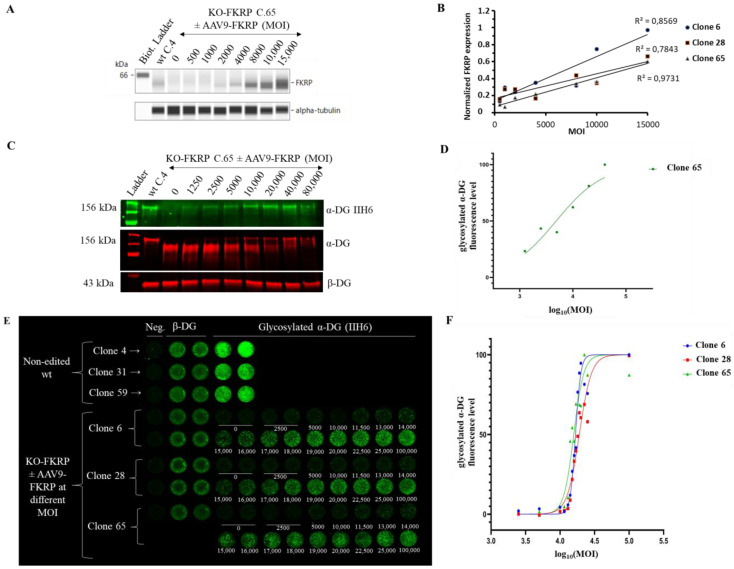
Restoration of glycosylated α-DG in KO-FKRP myotubes after rAAV-FKRP transduction. (**A**) Expression of the FKRP protein by Simple Western from a non-edited wt clone (wt C.4) and KO-FKRP clone 65 (C.65) transduced with rAAV-FKRP at different MOI doses: 0/500/1000/2000/4000/8000/10,000/15,000. α-tubulin was used as a housekeeping protein. The Western blot of clone 6 is representative of the edited clones. (**B**) Quantification of FKRP protein expression, normalized by α-tubulin, in KO-FKRP clones 6, 28, and 65, transduced with rAAV-FKRP at different MOI doses: 0/500/1000/2000/4000/8000/10,000/15,000. (**C**) Expression of the α-DG glycosylated using IIH6 antibody and the total α-DG by Western blot in KO-FKRP clone 65 (C.65) transduced with rAAV-FKRP at different MOI doses: 0/1250/2500/5000/10,000/20,000/40,000/80,000. β-DG was used as a housekeeping protein. (**D**) Western blot quantification of α-DG IIH6 expression, normalized by β-DG, from KO-FKRP clone 65. (**E**) Image of On-Cell Western results showing the expression of α-DG glycosylated (α-DG IIH6) and β-DG in wt clones (clone 4, clone 31, and clone 59) and KO-FKRP (clone 6, clone 28, and clone 65) myotubes. The KO-FKRP clones were transduced with rAAV-FKRP at 15 different MOI doses: 0/2500/5000/10,000/11,500/13,000/14,000/15,000/16,000/17,000/18,000/19,000/20,000/22,500/25,000/100,000. The green fluorescent staining is representative of the level of protein in each well. β-DG was used as a housekeeping protein. Neg. = negative control. (**F**) Quantification of α-DG IIH6 expression in KO-FKRP clones (clone 6, clone 28, and clone 65). The half maximal effective concentration (EC_50_) was determined for each clone.

To evaluate the biological activity of the FKRP gene therapy product, we next used OCW to study the restoration of α-DG glycosylation in KO-FKRP clones 6, 28, and 65 (Figure 5) after transduction with the GNT0006/ATA-100 vector.

The expression of the FKRP protein is increased in a MOI dose-dependent manner in all clones (Figure 5A,B). rAAV-FKRP treatment results also in a dose-dependent increase in α-DG glycosylation, as shown by the Western blot method, and leads to the progressive rescue of the molecular weight of the core α-DG (Figure 5C,D). We next demonstrated via the OCW method the similar MOI dose-dependent restoration of the glycosylated α-DG (MOI: 2500/5000/10,000/11,500/13,000/14,000/15,000/16,000/17,000/18,000/19,000/20,000/22,500/25,000/100,000) in all KO-FKRP clones (Figure 5E,F). Importantly, the half maximal effective concentration (EC_50_) for each clone (clone 6 EC_50_: 16,726; clone 28 EC_50_: 18,865; and clone 65 EC_50_: 15,773) illustrates the similar behavior of the edited clones and the accuracy of the OCW method. Importantly, EC_50_ can be easily assessed by OCW, while the determination of the EC_50_ is more difficult by Western blot analyses, limited by low throughput (Figure 5D). 

Overall, we developed a quantitative and high-throughput method to analyze the restoration of α-DG glycosylation after transduction by FKRP gene therapy products in a KO-FKRP muscle cell model.

### 3.5. Proof of Concept of rAAV Batch-to-Batch Comparison

**Figure 6:**
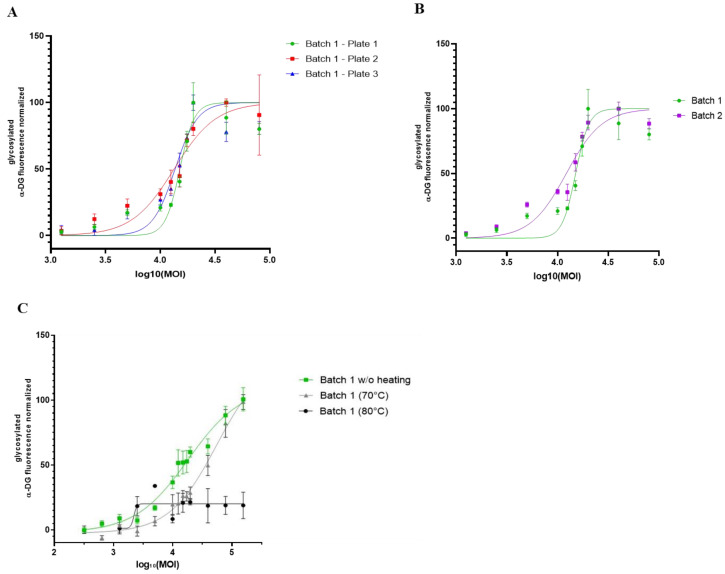
Comparison of different rAAV-FKRP vector batches by On-Cell Western method. (**A**) Quantification of glycosylated α-DG IIH6 expression in KO-FKRP clone 65 transduced with rAAV-FKRP at 10 different MOI doses: 0/1250/2500/5000/10,000/12,500/15,000/17,500/20,000/40,000/80,000 (n = 3 per MOI). The signal was normalized by the housekeeping protein β-DG. The half maximal effective concentration (EC_50_) was determined for each curve, representing three independent assays. (**B**) Quantification of glycosylated α-DG IIH6 expression in KO-FKRP clone 65 transduced with rAAV-FKRP at 10 different MOI doses: 0/1250/2500/5000/10,000/12,500/15,000/17,500/20,000/40,000/80,000 (n = 3 per MOI). The signal was normalized by the housekeeping protein β-DG. The half maximal effective concentration (EC_50_) was determined for each curve, representing data from 2 different rAAV batches. (**C**) Quantification of glycosylated α-DG IIH6 expression in KO-FKRP clone 65 transduced with rAAV-FKRP at 10 different MOI doses: 0/1250/2500/5000/10,000/12,500/15,000/17,500/20,000/40,000/80,000 (n = 3 per MOI). The signal was normalized by the housekeeping protein β-DG. The half maximal effective concentration (EC_50_) was determined for each curve, representing data from batch 1 without heating and with heating at 70 °C or 80 °C.

Combining the OCW and the KO-FKRP muscle cell model, we finally investigated the robustness and the ability of this methodology to compare the efficiency of different rAAV batches (Figure 6A). We used clone 65 for further experiments based on its KO-FKRP homozygous state. We first treated KO-FKRP myotubes from this clone with different MOI doses of the GNT0006/ATA-100 product (batch 1) and quantified, in three independent studies, the α-DG glycosylation signal normalized by the β-DG staining. All EC_50_ values were very close in the three independent experiments, with 15,053, 13,299, and 13,607 MOI, respectively. Similarly, KO-FKRP myotubes (clone 65) were transduced with a second batch of the GNT0006/ATA-100 product (batch 2) and the α-DG glycosylation was quantified (Figure 6B). The quantification of the normalized glycosylated α-DG glycosylation and the determination of the EC_50_ demonstrated a very close EC_50_ between batch 2 (12,204 MOI) and batch 1 (15,053 MOI). To demonstrate the ability of the assay to evaluate the potency batch-to-batch, we transduced myotubes with denatured products, the GNT0006/ATA-100 product heated at +70 °C or +80 °C for 10 min (Figure 6C). As described in Figure 6C, when the rAAV-FKRP vector is denatured by heating at +70 °C for 10 min, the effect on the glycosylation of α-DG is less effective compared to the control one (batch 1 without heating EC_50_: 15,424 versus batch 1 (70 °C) EC_50_: 32,469). Indeed, this treatment only reduces the potency of the viral gene therapy vector, whereas higher heating, at +80 °C, does not provide any restoration of α-DG glycosylation (Figure 6C). 

In conclusion, we developed a proof of concept for a cell-based assay allowing a batch-to-batch comparison of our rAAV-FKRP products in a dose–response manner using a high-throughput OCW method.

## 4. Discussion

In the present study, using the CRISPR-Cas9 gene editing system, we generated an in vitro KO-FKRP muscle cell model without any glycosylation of α-DG. Based on this newly edited model, we developed a quantitative and high-throughput cell-based potency assay to evaluate the biological activity of our FKRP transgene in the restoration of α-DG glycosylation and potentially assess the potency of other FKRP gene therapy products.

Potency assay development, qualification, and validation for gene therapy products is challenging [22]. Whereas the analytical methods must follow the ICH(Q2)R1 guidelines for the quality control of gene therapy products using different in vitro, in vivo, or multiple models, the potency assay aims to represent the MoA of the investigational medicinal product. Nevertheless, for most of the developmental therapeutic solutions, the MoAs are complex and not fully characterized [21]. Indeed, for such complex products with non-fully characterized and convoluted MoAs, more than one assay is required to develop a robust potency test [21]. In the gene therapy field [37] and for vaccines [38], some potency assays have been developed using in vivo models, but, lately, considering the variability of the results with animal models [39] and the need to apply the 3Rs (Replacement, Reduction, and Refinement) as recommended by regulatory agencies [40], pharmaceutical companies have moved on to in vitro models. Importantly, in vitro potency tests are less time-consuming, less labor-intensive, and cheaper than in vivo models. Successful potency assays were previously developed in liver [41] and retinal gene therapies [42].

Here, we developed and proposed an in vitro potency assay based on the most characterized MoA of the FKRP protein, the glycosylation of α-DG [23,24]. FKRP mutations are responsible for LGMDR9, a form of autosomal recessive Limb-Girdle Muscular Dystrophy. To counteract this neuromuscular disorder, an rAAV gene therapy encompassing the full-length human FKRP gene (GNT0006/ATA-100 product) was developed and, following extensive pre-clinical development [20], recently entered phase 1/2 clinical trials. With the objective of validating commercial rAAV-FKRP batches and complying with regulatory agencies’ obligations, a robust in vitro model and subsequent cell-based potency assays are required. Nevertheless, in some cases, a relevant cellular model is not available and an appropriate cell line with the desired genetic context has to be generated. Previously, several edited cell lines, such as the HEK293 knock-out for the SLC35A1 gene [43], or genetically modified reporter cell lines were produced to develop potency assays [44,45,46,47].

Taking advantage of the CRISPR-Cas9 system, we generated a KO-FKRP muscle cell line with the appropriate context to develop a robust potency assay and evaluate the biological activity of our rAAV-FKRP gene therapy product. Importantly, whereas FKRP-null mutations are lethal to embryos, LGMDR9 patients suffering from genetic defects in the FKRP gene present a partially functional protein leading to residual enzymatic activity on the glycosylation of α-DG [48,49]. In agreement with previous studies, our data demonstrate a similar level of glycosylated α-DG between wt and LGMDR9 myotubes (Figure S2A), making the assessment of a therapeutic solution complicated. Whereas rAAV-FKRP treatment in the LGMDR9 knock-in mouse model [20] leads to significantly increased α-DG glycosylation, resulting in histological and functional improvements, FKRP gene therapy produces only a slight and non-reproducible increase in glycosylation in LGMDR9 patients’ cells (data not shown), highlighting the limitations of the current in vitro model as well as the relevance of a newly generated KO-FKRP cell line without any interference of the basal level of α-DG glycosylation to develop a strong potency assay. The new in vitro model may also pave the way for further pre-clinical studies to gain insight into FKRP functions and associated dysregulated pathways.

We subsequently developed a valuable potency in vitro assay based on the OCW technology to assess a FKRP gene therapy product. After CRISPR-Cas9 transfection in wt myoblasts and clonal selection, we isolated and selected clones presenting gene editing on the two FKRP alleles. These edited clones demonstrated no FKRP expression or glycosylation of α-DG while retaining strong myogenic differentiation capacity. After FKRP gene therapy treatment, α-DG glycosylation was restored in an MOI-dose-dependent manner using OCW technology. Thus far, the quantification of the α-DG glycosylation has been mainly determined by protein immunoblotting [17,20,50], limited by low throughput and sometimes by complicated interpretation and reproducibility [51]. In contrast, the OCW methodology benefits from high-throughput quantification and allows batch-to-batch comparison. Similar high-throughput methods using the In-Cell Western (ICW) technology were previously successfully developed to quantify dystrophin to evaluate exon skipping efficiency after antisense oligonucleotide treatment in Duchenne Muscular Dystrophy (DMD) patients’ myotubes [52,53]. Therefore, the ICW and OCW technologies offer new perspectives and high-throughput solutions to screen pre-clinical drugs.

Whereas this work presents a proof of concept of a cell-based potency assay to assess an rAAV-FKRP gene therapy product, the validation of this analytical procedure is yet to be consolidated following the ICHQ2(R1) guidelines, and several parameters, such as the accuracy, the detection threshold, and the precision, require rigorous evaluation to determine the acceptance criteria for each characteristic [54]. Furthermore, the great versatility of the CRISPR-Cas9 system also comes with several limitations, such as potential off-target effects. A preliminary in silico off-target analysis indicated, among the top 10 off-targets for each sgRNA used, four predicted exonic off-targets: PGAM2, C6orf226, CD22, and PYGM (Table S1). While none of these genes are directly involved in proliferation and differentiation processes, as well as in FKRP regulation and α-DG glycosylation, further studies are required to fully validate the final KO-FKRP clone selected.

## 5. Conclusions

In summary, our study proposed the generation of an in vitro KO-FKRP model fully depleted of α-DG glycosylation, necessary for the subsequent development of an analytical method aiming to quantify α-DG glycosylation to validate a FKRP gene therapy product. This proof-of-principle potency assay allows the study of the biological activity of our rAAV-FKRP vector in an MOI-dose-dependent manner and the comparison, in a high-throughput approach, of the production batches against a reference material, addressing regulatory obligations throughout pharmaceutical and clinical development. Whereas such tools may serve to decipher new mechanisms in LGMDR9 pathology and gain insight into FKRP regulation, the generation of the newly KO-FKRP in vitro model combined with the OCW high-throughput methodology may be used to assess temperature stability, support the future optimization of the FKRP gene therapy with newly optimized rAAV capsid variants [55], and possibly serve as a screening tool to study combinatory solutions with small drugs [56,57].

## Data Availability

The data are available from the authors upon request.

## Supplementary Materials

The following supporting information can be downloaded at: https://www.mdpi.com/article/10.3390/cells12202444/s1, Figure S1: Gene editing efficiency and frequency of InDels determined using the ICE software; Figure S2: Expression of glycosylated α-DG in the selection of 16 clones; Figure S3: Expression of glycosylated α-DG in WT and FKRP immortalized cell lines; Figure S4: Myotube differentiation observed by microscopic analysis and immunofluorescence staining in edited clones; Table S1: In silico analysis of off-targets with CRISPOR TEFOR.
