# Supplementary material for: CRISPR-Cas9 KO Cell Line Generation and Development of a Cell-Based Potency Assay for rAAV-FKRP Gene Therapy
Source: Cells. 2023 Oct 12;12(20):2444. doi: 10.3390/cells12202444 (PMC10604961; doi:10.3390/cells12202444)
Supplement: Supplementary file 1 [file cells-12-02444-s001.zip › cells-2554502-supplementary.pdf]

Supplemental Materials

CRISPR-Cas9 KO Cell Line Generation and Development of a Cell-Based Potency Assay for rAAV-FKRP Gene Therapy

Marine Geoffroy [1,2,*], Louna Pili [1,2], Valentina Buffa [1,2], Maëlle Caroff [1,2], Anne Bigot [3], Evelyne Gicquel [1,2],
Grégory Rouby [1,2], Isabelle Richard [1,2,4] and Romain Fragnoud [1,2,*]

[1] Généthon, 91000 Evry-Courcouronnes, France
[2] Université Paris-Saclay/Université Evry, INSERM, Généthon, Integrare Research Unit, UMR_S951,
91000 Evry, France
[3] Institut de Myologie, Université Pierre et Marie Curie Paris 6, UM76 Univ. Paris 6/U974 UMR7215, CNRS Pitié-Salpétrière-INSERM, UMRS 974, 75000 Paris, France
[4] Atamyo Therapeutics, F-91000 Evry, France
* Correspondence: marine.geoffroy@sqy-synthena.com (M.G.); rfragnoud@genethon.fr (R.F.);
Tel.: +33-(0)169471042 (R.F.)

A

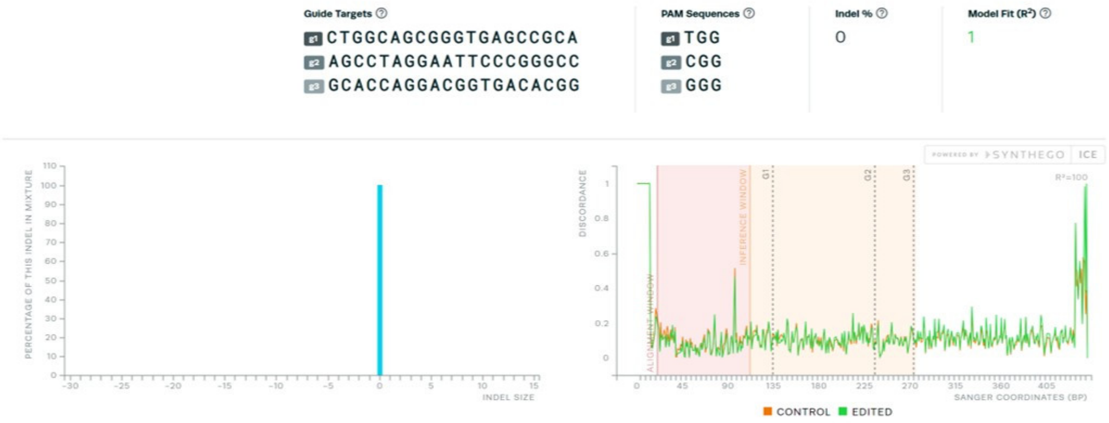
Guide Targets
CTGGCAGCGGGTGAGCCGCA
AGCCTAGGAATTCCCGGGCC
GCACCAGGACGGTGACACGG
PAM Sequences
TGG
CGG
GGG
Indel %
0
Model Fit (R²)
1
POWERED BY SYNTHEGO ICE
PERCENTAGE OF THIS INDEL IN MIXTURE
INDEL SIZE
DISCORDANCE
ALIGNMENT WINDOW
INFERENCE WINDOW
G1
G2
G3
SANGER COORDINATES (BP)
CONTROL
EDITED

B

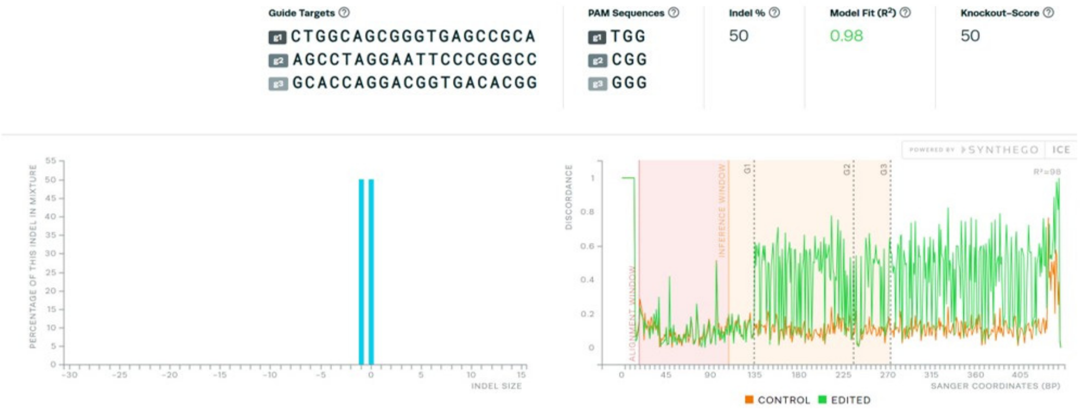
Guide Targets
CTGGCAGCGGGTGAGCCGCA
AGCCTAGGAATTCCCGGGCC
GCACCAGGACGGTGACACGG
PAM Sequences
TGG
CGG
GGG
Indel %
50
Model Fit (R²)
0.98
Knockout-Score
50
POWERED BY SYNTHEGO ICE
PERCENTAGE OF THIS INDEL IN MIXTURE
INDEL SIZE
DISCORDANCE
ALIGNMENT WINDOW
INFERENCE WINDOW
G1
G2
G3
SANGER COORDINATES (BP)
CONTROL
EDITED

C

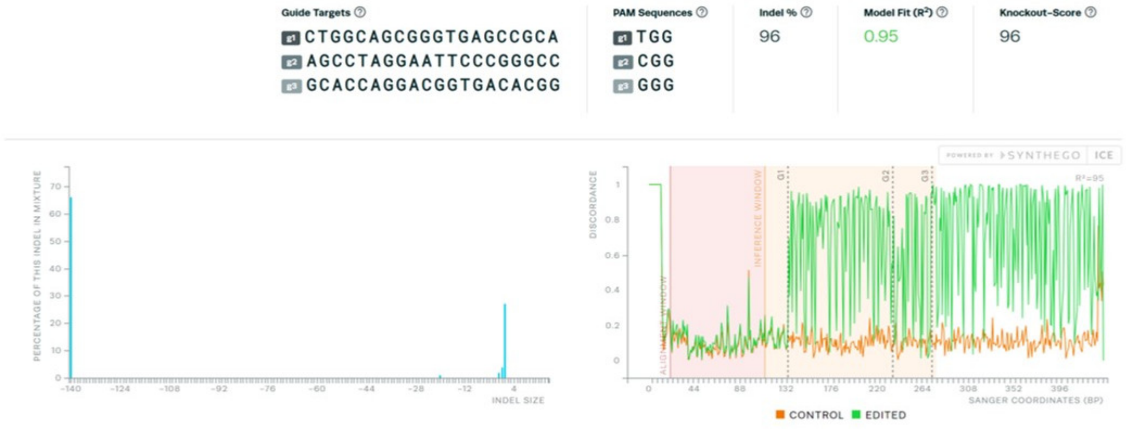
Guide Targets
CTGGCAGCGGGTGAGCCGCA
AGCCTAGGAATTCCCGGGCC
GCACCAGGACGGTGACACGG
PAM Sequences
TGG
CGG
GGG
Indel %
96
Model Fit (R²)
0.95
Knockout-Score
96
POWERED BY SYNTHEGO ICE
PERCENTAGE OF THIS INDEL IN MIXTURE
INDEL SIZE
DISCORDANCE
ALIGNMENT WINDOW
INFERENCE WINDOW
G1
G2
G3
SANGER COORDINATES (BP)
CONTROL
EDITED

D

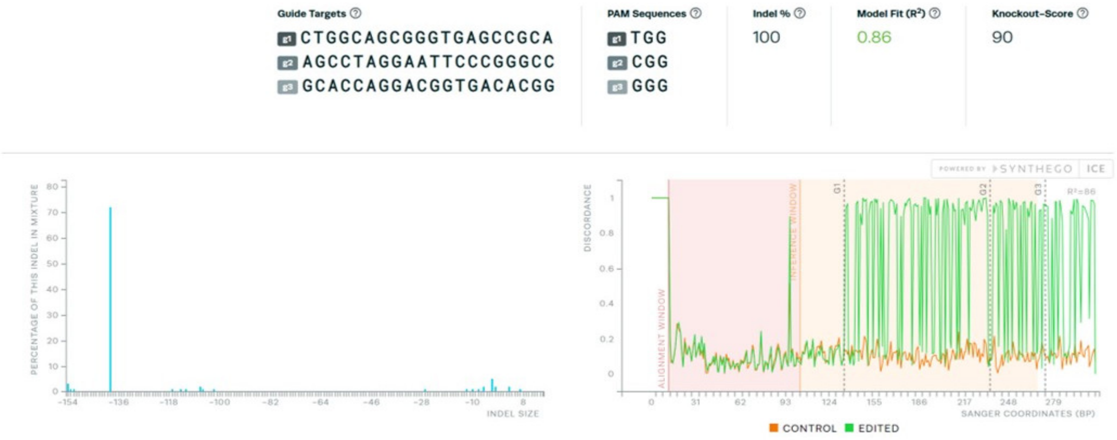
Guide Targets
CTGGCAGCGGGTGAGCCGCA
AGCCTAGGAATTCCCGGGCC
GCACCAGGACGGTGACACGG
PAM Sequences
TGG
CGG
GGG
Indel %
100
Model Fit (R²)
0.86
Knockout-Score
90
POWERED BY SYNTHEGO ICE
PERCENTAGE OF THIS INDEL IN MIXTURE
INDEL SIZE
DISCORDANCE
ALIGNMENT WINDOW
INFERENCE WINDOW
G1
G2
G3
SANGER COORDINATES (BP)
CONTROL
EDITED

Figure S1. Gene editing efficiency and frequency of InDels determined using the ICE Software. **(A)** Representation of InDels in the non-edited wt clones (X/X); Top: sgRNAs sequences used and percentage of InDels for the mixture of sgRNAs. Bottom left: ICE InDels profiles generated by the sgRNAs. Bottom right: Comparison of the reference non-edited sequence (control in orange) and the non-edited wt clone (edited in green). **(B)** Representation of InDels in the edited heterozygous clones (X/A); Top: sgRNAs sequences used and percentage of InDels for the mixture of sgRNAs. Bottom left: ICE InDels profiles generated by the sgRNAs. Bottom right: Comparison of the reference non-edited sequence (control in orange) and the edited heterozygous clone (edited in green). **(C)** Representation of InDels in the edited compound heterozygous clones (A'/A); Top: sgRNAs sequences used and percentage of InDels for the mixture of sgRNAs. Bottom left: ICE InDels profiles generated by the sgRNAs. Bottom right: Comparison of the reference sequence non-edited (control in orange) and the edited compound heterozygous clone (edited in green). **(D)** Representation of InDels in the edited homozygous clones (A/A); Top: sgRNAs sequences used and percentage of InDels for the mixture of sgRNAs. Bottom left: ICE InDels profiles generated by the sgRNAs. Bottom right: Comparison of the reference non-edited sequence (control in orange) and the edited homozygous clone (edited in green).

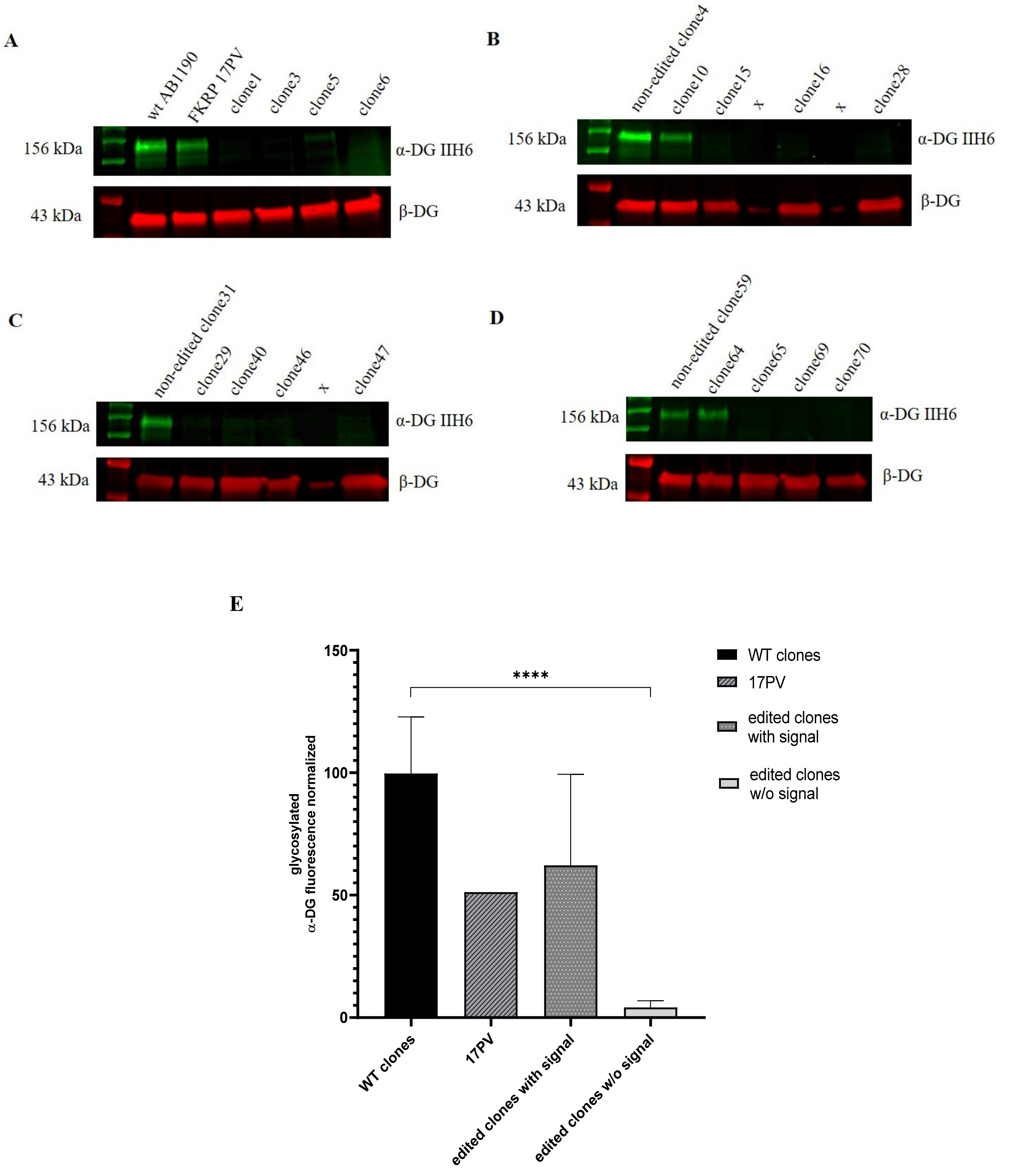

Figure S2. Expression of glycosylated α-DG in the selection of 16 clones. **(A)** Expression of the glycosylated α-DG using IIH6 antibody by western blot from the original wt myotubes (wt AB1190), the immortalized LGMDR9 patients myotubes (FKRP 17PV), and the edited clones (clone 1, clone 3, clone 5 and clone 6). β-DG was used as housekeeping protein. **(B)** Expression of the glycosylated α-DG using IIH6 antibody by western blot from the non-edited clone 4 and the edited clones (clone 10, clone 15, clone 16 and clone 28). β-DG was used as housekeeping protein. The "x" corresponds to an empty well. **(C)** Expression of the glycosylated α-DG using IIH6 antibody by western blot from the non-edited clone 31 and the edited clones (clone 29, clone 40, clone 46 and clone 47). β-DG was used as housekeeping protein. The "x" corresponds to an empty well. **(D)** Expression of the glycosylated α-DG using IIH6 antibody by western blot from the non-edited clone 59 and the edited clones (clone 64, clone 65, clone 69 and clone 70). β-DG was used as housekeeping protein. **(E)**

Quantification of glycosylated α-DG normalized in 4 groups: WT/non edited clone (AB1190/clone 4, 31 and 59), FKRP 17PV cell line, edited clones with glycosylated α-DG signal (clones 5, 10 and 64), edited clones with no glycosylated α-DG signal (clones 1, 3, 6, 15, 16, 28, 29, 40, 47, 65, 69 and 70). **** p-value < 0.0001 following an unpaired t-test.

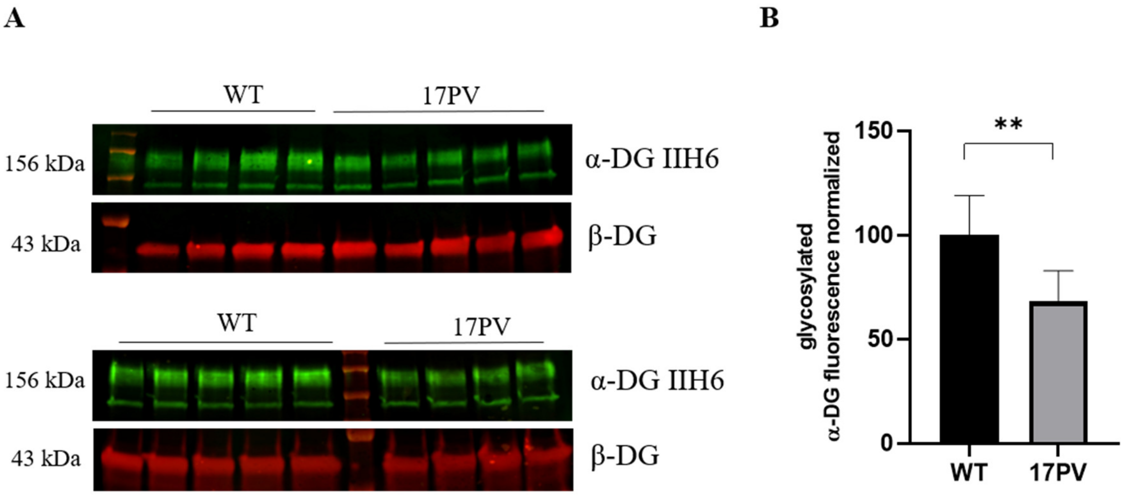

Figure S3. Expression of glycosylated α-DG in WT and FKRP immortalized cell lines. **(A)** Expression of the glycosylated α-DG using IIH6 antibody by western blot from the original wt myotubes (wt AB1190) and the immortalized LGMDR9 patients myotubes (FKRP 17PV). Two different experiments were performed. **(B)** Quantification and normalization of glycosylated α-DG normalized (n=9 per cell lines). ** p-value = 0.0011 following an unpaired t-test.

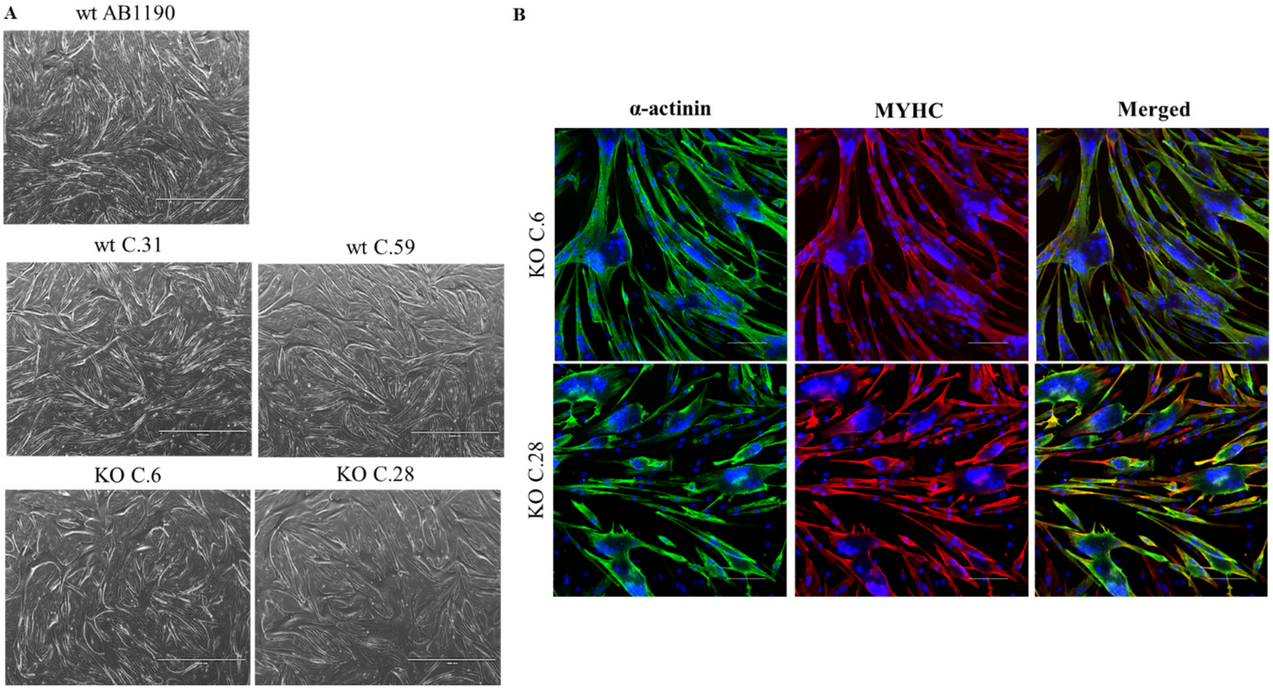

Figure S4. Myotube differentiation observed by microscopic analysis and immunofluorescence staining in edited clones. (A) Brightfield pictures of the original wt cells (AB1190), the non-edited wt clones (wt C.31 and wt C.59) and the edited wt clones (KO C.6 and KO C.28) at myotube stage. Scale bars = 1,000 µm. (B) Immunofluorescence staining (α-actinin and MYHC) of the edited clones (KO C.6 and KO C.28). Cell nuclei were labelled with DAPI dye. Scale bars = 100 µm.

Table S1. *In silico* analysis of off-targets with CRISPOR TEFOR **(A)** Top 10 locus of off-targets for sgRNA1. **(B)** Top 10 locus of off-targets for sgRNA2. **(C)** Top 10 locus of off-targets for sgRNA3.

A

Locus	mismatchCount	cfdOfftargetScore	chrom	start	end	strand
**FKRP**	**0**	**100**	**chr19**	**46755455**	**46755477**	**-**
intergenic:RP11-1151B14.2-RP11-1151B14.3	4	0.51	chr18	56112872	56112894	-
intergenic:AL136967.1-RP11-328M4.2	3	0.46	chr6	41384109	41384131	+
intron:ZNF862	4	0.40	chr7	149551791	149551813	-
intergenic:RP11-128M1.1-TGM3	3	0.34	chr20	2268800	2268822	+
exon:DBNL/PGAM2/AC017116.11	4	0.34	chr7	44101156	44101178	-
intergenic:LMBR1-RNF32	3	0.31	chr7	156462214	156462236	+
intron:MYO18B	4	0.30	chr22	26145944	26145966	+
intergenic:RP11-386G21.2-XKR4	4	0.30	chr8	56083410	56083432	+
intergenic:RP11-761N21.1-RP11-520A21.1	4	0.29	chr3	40950300	40950322	-
intergenic:RXRA-RP11-473E2.2	4	0.28	chr9	137393918	137393940	-

B

Locus	mismatchCount	cfdOfftargetScore	chrom	start	end	strand
**FKRP**	**0**	**100**	**chr19**	**46755556**	**46755578**	**+**
intergenic:CTC-461H2.2-RHPN2	4	0.71	chr19	33554035	33554057	+
intergenic:KCNN3-PMVK	3	0.67	chr1	154845237	154845259	-
lnc:AC006116.17/ZSCAN5A/Y_RNA	3	0.62	chr19	56826681	56826703	+
intergenic:RNU7-145P-RP11-142I20.1	4	0.53	chr18	38950440	38950462	-
intergenic:RPS23P3-RNU6-699P	4	0.50	chr4	67430059	67430081	+
lnc:RP11-50E11.2/RP11-50E11.3	4	0.41	chr10	52397448	52397470	-
intron:DFNB31	4	0.40	chr9	117170569	117170591	-
intergenic:COMP-UPF1	4	0.39	chr19	18924078	18924100	-
exon:C6orf226	4	0.33	chr6	42858415	42858437	-
exon:CD22	3	0.30	chr19	35820497	35820519	+

C

Locus	mismatchCount	cfdOfftargetScore	chrom	start	end	strand
**FKRP**	**0**	**100**	**chr19**	**46755594**	**46755616**	**-**
exon:PYGM	4	0.57	chr11	64518824	64518846	+
intron:RP11-890B15.2	4	0.54	chr11	130723063	130723085	-
intron:GPR56	4	0.39	chr16	57692790	57692812	+
intergenic:SNAP25-AS1-SDAD1P2	4	0.37	chr20	10360573	10360595	+
intron:AGAP3	3	0.33	chr7	150805390	150805412	-
intergenic:MDH2-AC005077.14	4	0.32	chr7	75698884	75698906	+
intron:CUX1	4	0.31	chr7	101818983	101819005	-
intron:AC008271.1	4	0.31	chr2	15831614	15831636	-
intergenic:RP11-324D17.2-RP11-324D17.1	4	0.30	chr16	54276113	54276135	-
intergenic:VPS37D-DNAJC30/WBSCR22	4	0.26	chr7	73090050	73090072	+
